# Familial summer-type hypersensitivity pneumonitis in Japan: two case reports and review of the literature

**DOI:** 10.1186/1756-0500-6-371

**Published:** 2013-09-13

**Authors:** Akira Nakajima, Takeshi Saraya, Takeshi Mori, Reiko Ikeda, Takashi Sugita, Takayasu Watanabe, Masachika Fujiwara, Hajime Takizawa, Hajime Goto

**Affiliations:** 1 Department of Respiratory Medicine, Kyorin University School of Medicine, 6-20-2 Shinkawa, 181-8611, Mitaka City, Tokyo, Japan; 2 Division of Hematology, Department of Internal Medicine, Juntendo University School of Medicine, 2-1-1 Hongou, 113-8421, Bunkyo ward, Tokyo, Japan; 3 Department of Microbiology, Meiji Pharmaceutical Universitys,2-522-1 Noshio, 204-0004, Kiyose city, Tokyo, Japan; 4 Department of Pathology, Kyorin University School of Medicine, 6-20-2 Shinkawa, 181-8611, Mitaka City, Tokyo, Japan

**Keywords:** Familial summer-type hypersensitivity pneumonitis, Climate, Geography, *Trichosporon* species, Environmental factor

## Abstract

**Background:**

Hypersensitivity pneumonitis is defined as an allergic lung disease that occurs in response to inhalation of fungal antigens, bacterial antigens, chemicals, dusts, or animal proteins. The incidence of summer-type hypersensitivity pneumonitis is higher in the summer season, especially in Japan, due to the influence of the hot and humid environment and the common style of wood house or old concrete condominiums.

**Case presentation:**

The present report describes a case of a middle-aged married couple who lived in the same house and who simultaneously suffered from summer-type hypersensitivity pneumonitis. This report analyzes these two cases in terms of environmental research and its microbiological, radiological, and pathological aspects. This case report is followed by a review of family occurrences of summer-type hypersensitivity pneumonitis from 22 studies with a total of 49 patients (including the two present cases) in Japan.

**Conclusion:**

Summer-type hypersensitivity pneumonitis may be unrecognized and misdiagnosed as pneumonia or other respiratory diseases. A greater understanding of the clinical, pathologic, and environmental features of summer-type hypersensitivity pneumonitis might help improve diagnosis and delivery of appropriate management for this condition.

## Background

Summer-type hypersensitivity pneumonitis (SHP) is a form of hypersensitivity pneumonitis caused by inhalation of *Trichosporon asahii* or *mucoides* during a hot and humid summer season. SHP has been reported in Japan since 1973 [1,2] and occasionally familial SHP has been described. However, no study has performed a review of the relevant literature so far, and the largest study described by Ando et al. did not clarify the detail of clinical findings associated with familial SHP [1]. Herein, we present two cases of familial SHP and a review of 49 patients in Japan.

## Case presentation

**Figure 1:**
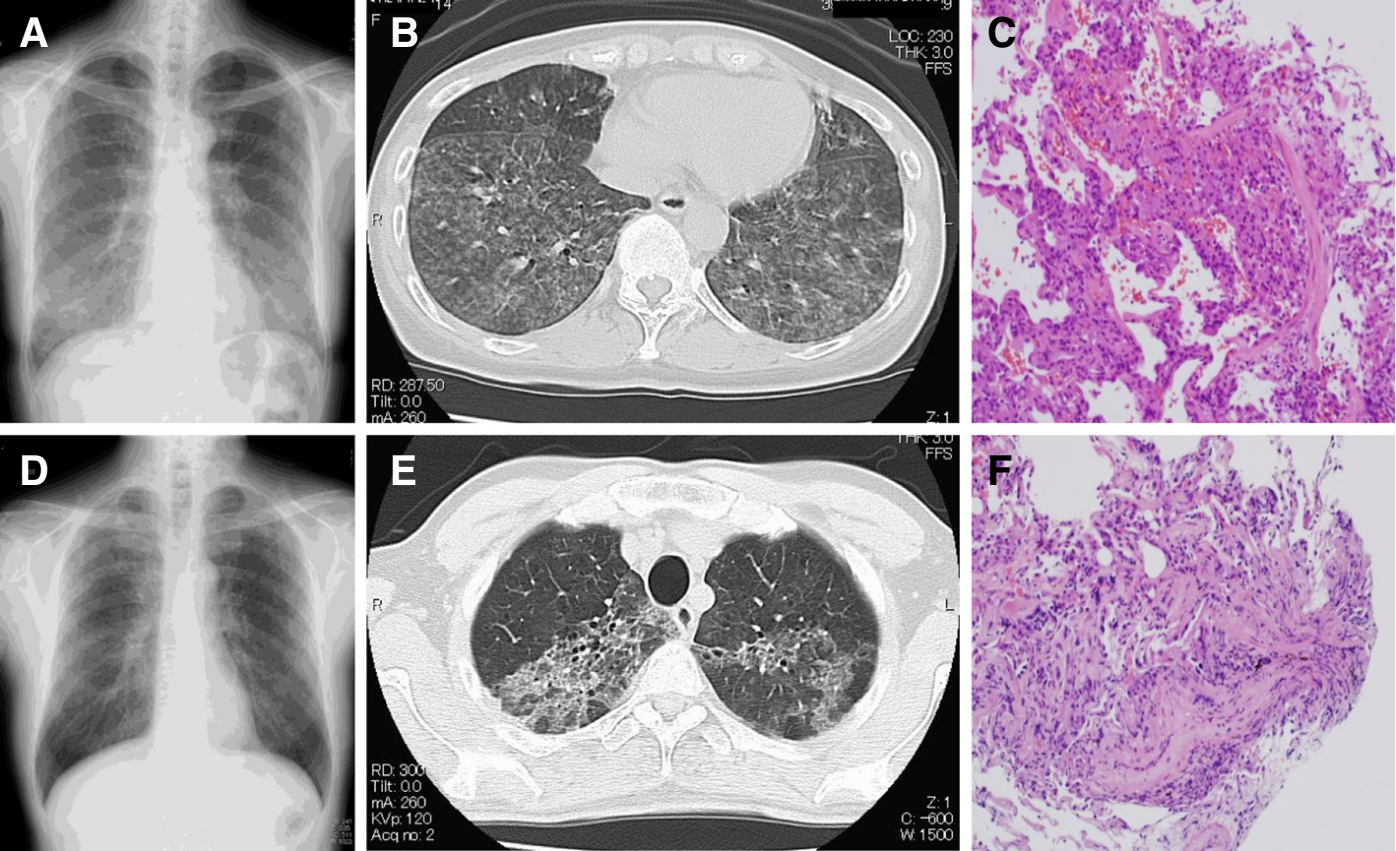
**Radiological and pathological assessment for two patients.** (wife; A, B, and C): Chest x-ray on the day of admission shows mild infiltrates predominantly in the middle to lower lung fields with multiple scattered nodular lesions **(Panel A)**. Thoracic CT (computed tomography) taken on the same day confirms that these lesions correspond to ground-glass opacities and abundant centrilobular nodules, predominantly located in the bilateral lower lobes **(Panel B)**. Specimens obtained from transbronchial lung biopsy (TBLB) demonstrate organization within the peribronchial area with alveolitis, suggesting transbronchial spread **(Panel C)**. (husband; D, E, and F): Chest x-ray **(Panel D)** and thoracic CT **(Panel E)** show ground glass opacities in the upper lungs on both sides. The specimens obtained from TBLB at his local hospital reveals organizing tissue within the peribronchial area with alveolitis, suggesting transbronchial spread **(Panel F)**.

A 58-year-old married woman was admitted to our hospital in August 2012 with a chief complaint of persistent fever (38°C), gradually progressive dyspnea on effort, and productive cough for over 1 month. She noticed that her symptoms recurred whenever she stayed in her house, but disappeared when she was outdoors. She had no remarkable medical history. She was a current smoker with a history of 10 pack-years and worked as a caregiver. Her vital signs were as follows: blood pressure of 108/70 mmHg, pulse rate of 89 beats/minute, temperature of 37.7°C, respiratory rate of 20 breaths per minute, and oxygen saturation of 89% at room air. Physical examination was normal except for inspiratory late crackles in all lung fields bilaterally. Chest x-ray (Figure 1A) showed mild infiltration, predominantly in the middle to lower lung fields, with multiple scattered nodular lesions. Thoracic computed tomography (CT) (Figure 1B) revealed ground-glass opacities and abundant centrilobular nodules throughout both lungs, especially in bilateral lower lobes. Laboratory examination showed elevation of lactase dehydrogenase (291 IU/L), C-reactive protein (4.2 mg/dL), Krebs von den Lungen-6 (1270 IU/L), and surfactant protein D (135 IU/L) (Table 1). Serum anti-*Trichosporon asahii* antibody was positive by enzyme immunoassay (1.91), and biopsied specimens obtained from transbronchial lung biopsy (TBLB) showed organization within the peribronchial area, with alveolitis accompanying lymphocytic infiltration (Figure 1C), suggesting transbronchial spread. Based on these data, she was diagnosed with SHP. Her chief complaints and hypoxemia resolved spontaneously by the second hospital day, and she was discharged on the fifth hospital day to her home. Two weeks later, she was re-admitted to our hospital with a diagnosis of recurrent SHP. She was subsequently treated with oral prednisolone (0.8 mg/kg/day). After cleaning up her house with disinfectant and re-covering the floor, she was discharged again on the eighth hospital day with complete resolution of her symptoms. Human leukocyte antigen (HLA) typing showed DR4, A11, A31, B54, B62, DQ4, and DQ8.

**Table 1:** 49 Cases of familial summer type hypersensitivity pneumonitis reported in Japan

**Year**	**Age**	**Sex**	**Season**	**Smoking history**	***Tricosporon *antibody**	**Respiratory failure**	**Steroid therapy**	**Move**	**Relapse**	**Ref**
1982	43	F	7	N.D	N.D	--	+	--	+	5
	18	M	8	N.D	N.D	--	+	--	--	5
1984	39	F	8	N.D	N.D	+	--	--	--	5
	12	M	8	N.D	N.D	+	+	--	--	5
1985	45	M	8	N.D	N.D	+	--	--	--	5
	40	F	8	N.D	N.D	+	+	--	--	5
	15	M	8	N.D	N.D	+	--	--	--	5
1987	42	F	8	+	N.D	--	--	--	N.D	5
	41	M	7	+	N.D	+	--	--	N.D	5
	19	F	8	+	N.D	+	+	--	N.D	5
1990	59	M	8	+	N.D	N.D	+	--	--	5
	57	F	8	--	+	+	+	--	+	5
1992	35	F	7	N.D	+	N.D	--	+	--	5
	14	F	8	--	+	--	--	+	--	5
1994	36	F	8	N.D	N.D	+	--	+	--	5
	8	F	9	--	N.D	+	+	+	--	5
1996	39	M	9	+	--	--	+	--	+	5
	38	F	9	--	+	--	+	--	+	5
	15	F	8	--	+	+	+	--	+	5
1997	36	F	8	N.D	+	+	+	--	--	5
	13	M	8	--	+	+	+	--	--	5
1997	63	F	6	N.D	--	--	--	N.D	N.D	5
	65	M	7	N.D	--	--	--	N.D	N.D	5
1998	43	F	N.D	--	N.D	N.D	+	--	+	5
	14	M	8	--	+	+	--	--	--	5
2000	65	M	8	+	+	+	--	--	--	6
	2	M	9	--	+	N.D	--	+	--	6
2001	35	F	5	+	+	--	--	--	--	5
	9	F	5	--	+	+	--	--	--	5
2002	57	F	8	--	+	--	+	--	+	5
	57	M	8	--	+	+	+	--	+	5
2003	24	M	8	--	+	--	--	+	--	5
	24	M	11	+	+	--	--	+	--	5
2004	9	M	7	--	+	+	+	+	--	5
	7	F	8	--	+	N.D	--	+	--	5
2005	37	M	7	--	+	--	--	+	--	5
	10	F	7	--	+	N.D	--	+	--	5
2005	45	F	8	--	+	--	+	+	--	7
	51	M	7	--	+	--	+	+	--	7
2005	32	F	8	--	+	--	+	--	--	8
	64	M	8	+	--	--	+	--	--	8
2007	18	F	10	--	+	N.D	+	--	--	9
	42	F	9	--	+	N.D	+	--	--	9
2008	45	M	6	N.D	+	+	+	+	--	10
	43	F	6	N.D	+	--	+	+	--	10
2009	74	M	8	+	+	+	--	--	+	11
	53	F	7	+	+	--	--	--	+	11
2012	58	F	7	+	+	+	+	--	+	Our case
	63	M	7	+	+	+	-	--	+	Our case

*N.D* not determined.

The same patient’s husband was a 63-year-old previously healthy man who was admitted to his local hospital with chief complaints of productive cough, dyspnea on effort, and fever of 38°C lasting for a few weeks from the beginning of August 2012. He had a smoking history of 25 pack-years and was diagnosed with chronic obstructive pulmonary disease a few years previously. He worked as a stage director and had been in good health until then. The specimens obtained by TBLB (Figure 1F) performed at his local hospital showed organizing tissue within peribronchial area with alveolitis, suggesting bronchial spread. He was tentatively diagnosed with atypical pneumonia and was treated with antibiotics. His symptoms completely resolved within a week, and he was discharged to his home. However, on the day of discharge, 3 hours after returning his home, his symptoms recurred and he presented to our hospital. Initial examination indicated that he was quite ill; his vital signs showed a temperature of 38°C, and oxygen saturation has dropped to about 80% at room air. His physical examination showed inspiratory late crackles in the bilateral upper to middle lung fields. Chest x-ray (Figure 1D) and thoracic CT (Figure 1E) showed ground glass opacities in the upper lungs bilaterally. Based on his medical history together with the fact that his wife had already been diagnosed with SHP at the same time, he was diagnosed with SHP. This was confirmed by positive result of serum anti-*Trichosporon asahii* antibody on enzyme immunoassay (1.21), and he was successfully discharged to his son’s house on the seventh hospital day with no treatment. His HLA typing showed DR8, A2, A26, B35, B55, and DQ4.

**Figure 2:**
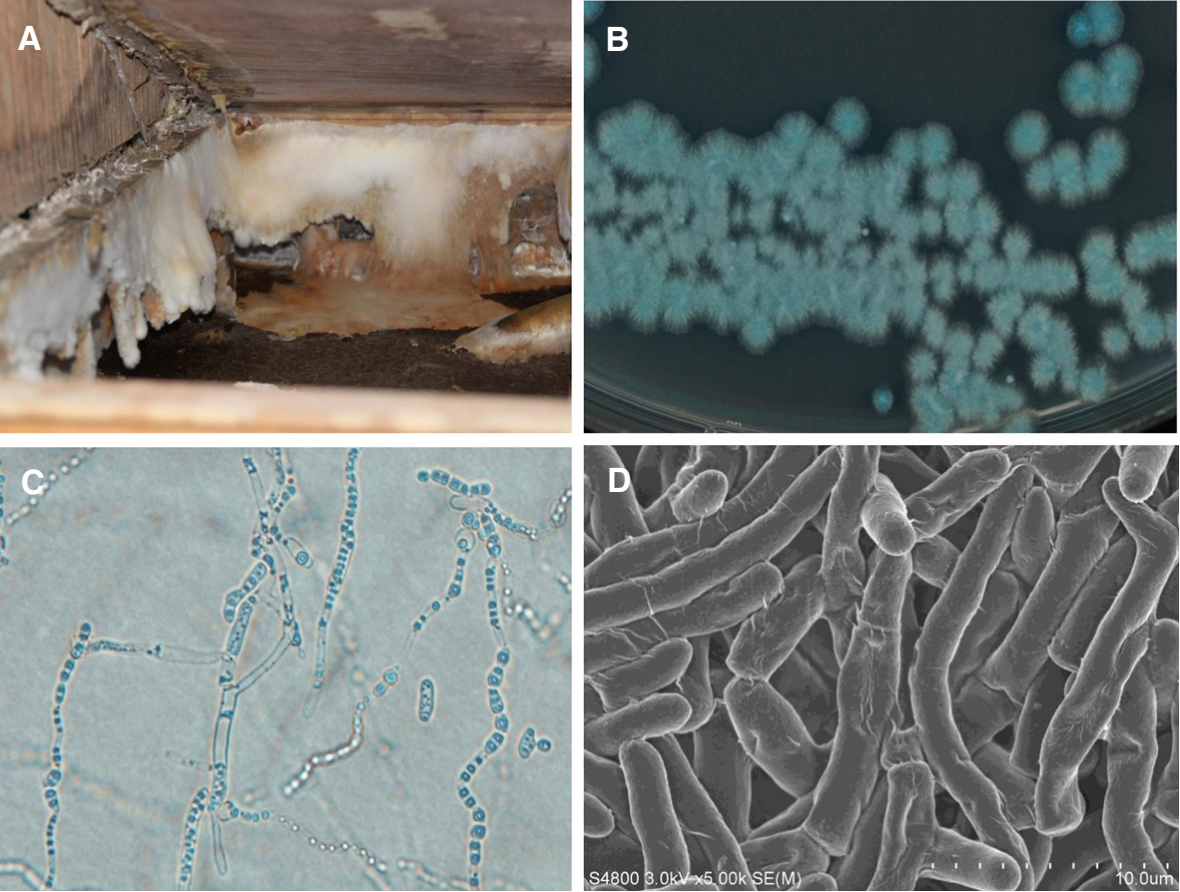
**Environmental research at the patient’s home and multidisciplinal identification for *Trichosporon asahii.*** Environmental research at the patients’ home showed tatami mats that were crumbled and in a decaying state with malodorous white matters beneath the mats **(Panel A).** On Sabouraud agar, multiple soft nodules of white piedra are cultured, suggesting *Trichisporon asahii***(Panel B)**. Light microscopy demonstrates the blue colored hyphae that disarticulate into rectangular arthroconidia with rounded ends **(Panel C)**. Electron microscopy clearly depicts those rounded ends with size up to 5 μm **(Panel D)**.

We conducted environmental research at the two patients’ house. Tatami mats (a floor fitting made of straw that are peculiar to Japan) were present and were noted to be crumbled and in a decaying state (Figure 2A). Beneath the tatami mats, numerous white-colored and malodorous particulates were recognized and culture of this material showed multiple soft nodules of white piedra on Sabouraud agar, suggestive of *Trichosporon asahii* (Figure 2B). Those white colonies produced blue colored hyphae that disarticulated into rectangular arthroconidia with rounded ends (Figure 2C) on light microscopy, measuring approximately 5 μm (Figure 2D) on electron microscopy. Further analysis on slide agglutination tests using *Trichosporon* antigen (type II; known as *T. asahii*) and serum from the two patients showed a positive anti-*T. asahii* titer (2X). Further, the agglutination test using the strain of *T. asahiii* isolated from their house (Figure 2) showed a positive result for anti-*T. asahii* titer both in the husband (4X) and in the wife (8X).

Fungal deoxyribonucleic acid (DNA) was extracted using the method of Makimura et al. [2] and the intergenic spacer (IGS) 1 region was sequenced according to the method of Sugita et al. [3]. Briefly, the *Trichosporon* IGS 1 region (approximately 500 bp) was amplified by polymerase chain reaction (PCR) using the oligonucleotide primers 26SF (5ÅL-ATCCTTTGCAGACGACTTGA-3ÅL) and 5SR (5ÅL-AGCTTGACTTCGCAGATCGG-3ÅL). The PCR products were sequenced with 26SF and 5SR using an ABI 3700 DNA sequencer with an ABI PRISM BigDye Terminator Cycle Sequencing kit (Applied Biosystems, CA, USA), according to the manufacturer’s instructions. Based on these methods, the white colonies on Sabouraud agar identified as *T. asahii*.

**Figure 3:**
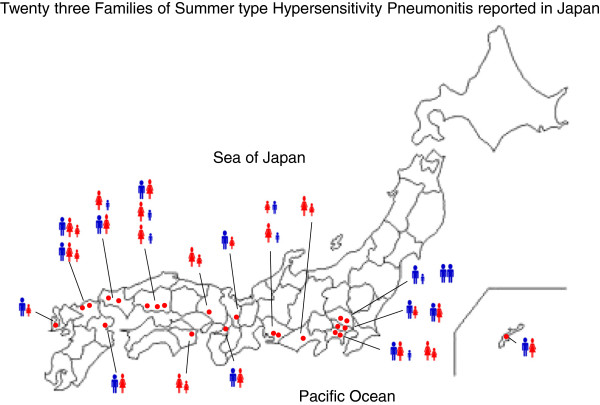
**Geographic distribution of the 49 cases of Japanese summer type hypersensitivity pneumonitis (SHP).** The disease is more common in the western and southern parts of the country and has a predilection for areas bordering the Pacific Ocean area when compared with areas facing the Sea of Japan. The northernmost site of the disease was in the Saitama Prefecture, located at a latitude of 36° north. ● indicates one SHP event. (blue human-shaped symbol) and (red human-shaped symbol) indicate male or female adult patients, respectively. (small blue human-shaped symbol) and (small red human- shaped symbol) indicate boy or girl patients who lived with their family. The map of Japan was obtained from the site (http://aoki2.si.gunma-u.ac.jp/map/map.html).

Using the PubMed database, we identified and reviewed 22 studies with a total of 49 patients (including the two present cases) of family occurrence of SHP in Japan (Table 1) [4-10]. The mean age of affected patients was 36.1 ± 15.9 (mean ± SD [SD: standard deviation]), the age range was from 2 to 74 years, and the male to female ratio was 22:27. Among the 49 patients, 20 patients were married couples, and the other 20 patients had blood relationships. Among the 49 patients, the number of smokers and non-smokers was 13 and 22, respectively. Information regarding smoking history was not obtained in the other 14 patients. Thirty-five patients were assessed for specific anti-*Trichosporon* spp. antibody, of whom 31 patients (88.5%) had positive results. The presence of acute respiratory failure (defined as oxygen saturation of less than 90%) was observed in 22 patients with SHP (44.9%). HLA phenotyping was assessed in 11 studies (data not shown), and the present two cases described SHP-sensitive HLA phenotypes in both the husband (A2) and the wife (A11, DQ8). The geographic distribution of the 49 cases of Japanese SHP showed a predilection for the western and southern parts of the country, and the condition was more common in prefectures bordering the Pacific Ocean than in those facing the Sea of Japan (Figure 3). The most northernmost case of the disease was in Saitama Prefecture, located at a latitude of 36°C north, and the area with the most cases (six studies; 13 patients) was the Kanto plain (Saitama and Kanagawa Prefectures and Tokyo area). Based on the annual records of the Japan Meteorological Agency, the average temperature and humidity at the time of onset in individual cases was 25.9°C ± 2.2 (mean ± SD.) and 74.1% ± 4.1 (mean ± SD.), respectively. Ninety percent of all cases of familial SHP in this study occurred from July to September, which corresponds to the after rainy season with high temperature and/or high humidity in Japan.

Review of the 49 cases shows the following management strategies: oral prednisolone only (n = 20, 43.5%), avoidance of fungal exposure and house cleaning (n = 11, 22.4%), moving to a new house (n = 10, 20.4%), moving to a new house and oral prednisolone (n = 6, 12.2%), and unknown (n = 2, 4.1%). Eleven patients (22.4%) experienced recurrence, all of whom stayed in the house without moving. No recurrence was noted in patients who moved to a new house.

## Discussion

Summer-type HP (SHP) is a form of HP caused by inhalation of *Trichosporon asahii* or *mucoides* during a hot and humid summer season. SHP accounts for three quarters of HP and has been reported in Japan since 1973 [1,11]. Familial SHP comprises 20 to 25% of cases of SHP [12-14], but no study has performed a review of the relevant literature so far. More than 90% of SHP patients had anti-*Trichosporon* antibodies or had a positive result for inhalation challenges of the antigen, as was seen in the present two cases. Review of the literature confirmed a geographic and climatic predilection for SHP depends on the environment, with the disease being more common according to an ambient temperature of 25-28°C and a relative humidity of 80% or more in the presence of damp wood [14], all of which represent better conditions for growth of *Trichosporon* spp.

Previous reports in Japan showed that HLA-DQw3 (DQ7, DQ8, DQ9) [15,16] and HLA-A11, A2 or DR9 [16] were genetic factors associated with SHP, as was seen in the present two cases. In addition, the disease was more common in non-smokers than in smokers [17], which may account for our result that the number of non-smokers was larger than that of smokers. Thus, development of familial SHP required the presence of environmental and genetic factors, including smoking and exposure to a large amount of inhalation antigens.

SHP may occur in any type of house, and approximately half of familial SHP patients suffered from acute respiratory failure in this study. Trichosporonosis occurs in areas where the mean maximum temperature is higher than 25°C for 6 months or more. Furthermore, some cases of familial SHP were reported in Korea [18] or southern Africa [19] where the temperature is relatively hot (>25°C) and the climate is humid (~80%), which suggests that some cases of SHP may be unrecognized and misdiagnosed as spontaneous pneumonia or other respiratory diseases. In this regard, a greater understanding the clinical, pathologic, and environmental features of SHP might help improve diagnosis and delivery of appropriate management for this condition.

## Conclusion

Summer-type hypersensitivity pneumonitis may be unrecognized and misdiagnosed as pneumonia or other respiratory diseases. A greater understanding of the clinical, pathologic, and environmental features of summer-type hypersensitivity pneumonitis might help improve diagnosis and delivery of appropriate management for this condition.

## Consent

Written informed consent was obtained from the patient for publication of this Case Report and any accompanying images. A copy of the written consent is available for review by the Editor-in-Chief of this journal.

## Abbreviations

SHP: Summer-type hypersensitivity pneumonitis.

## Competing interests

The authors declare that they have no competing interests.

## Authors’ contributions

AN and TS drafted the initial manuscript and modified it in reference to the other. HT and HG edited the manuscript. AN, TS, and TW were involved in diagnostics and treatment of the patient. MF analyzed pathological findings. TM, IR, and TS contributed to all laboratory examinations. All authors read and approved the final manuscript.
